# Ultrasonic-Assisted Fabrication and Stability Evaluation of Pepper Seed Protein Nanoemulsions

**DOI:** 10.3390/foods14162779

**Published:** 2025-08-10

**Authors:** Limin Wu, Mengmeng Wei, Ninghai Lu, Benguo Liu

**Affiliations:** 1 School of Plant Protection and Environment, Henan Institute of Science and Technology, Xinxiang 453003, China; zbw@hist.edu.cn (L.W.); gsninghai@hist.edu.cn (N.L.); 2 School of Food Science, Henan Institute of Science and Technology, Xinxiang 453003, China; 15236568854@163.com

**Keywords:** pepper seed protein isolate, nanoemulsion, ultrasonic emulsifying, optimization, stability

## Abstract

Pepper seeds, a key byproduct of pepper processing, are rich in high-quality plant proteins. This study investigated the structural and functional properties of pepper seed protein isolate (PSPI) and optimized the ultrasonic homogenization process for PSPI-based nanoemulsions using response surface methodology (RSM), followed by stability evaluation. The results showed that glutamic acid is the dominant amino acid in PSPI, with a molecular weight range of 10–55 kDa. Some protein subunits were interconnected via disulfide bonds. Functionally, PSPI had lower water-/oil-holding capacities but superior emulsifying activity compared to soy protein isolate (SPI). RSM optimization determined the optimal nanoemulsion parameters within experimental constraints: PSPI 0.53%, ultrasonic power 500 W, and ultrasonic time 130 s, yielding a nanoemulsion with a droplet size of 319 ± 2 nm, consistent with the theoretical prediction (318 nm). The nanoemulsion demonstrated stability under neutral-to-alkaline conditions (pH 7.0–9.0), high ionic strength (Na^+^ concentration ≤ 100 mM), and elevated temperatures (40–100 °C), without phase separation or aggregation. This work supports pepper seed protein utilization and provides insights for plant protein nanoemulsion production.

## 1. Introduction

Pepper (*Capsicum* spp.), an economically significant crop of the Solanaceae family, currently covers approximately 3.7 million hectares globally with an annual production exceeding 60 million tons, ranking as the world’s third most important vegetable crop [1]. Beyond its traditional use as a seasoning and vegetable, recent studies have revealed its remarkable bioactivities, including substantial medicinal potential in antitumor, antioxidant, and anti-obesity applications [2,3,4]. Among pepper processing byproducts, pepper seeds account for 30–60% of the total waste and are a valuable underutilized resource. Previous studies have demonstrated that pepper seeds contain significant amounts of bioactive compounds, including total phenolics (29.1 mg GAE/g), flavonoids (21.3 mg GAE/g), sterols, and saponins [5,6]. Notably, they exhibit potent ABTS radical scavenging capacity, demonstrating strong antioxidant properties [7]. Nutritionally, pepper seeds contain 13.7–28.33% protein [8,9], with a rich profile of essential amino acids (leucine, lysine, threonine, etc.). Importantly, their amino acid composition meets the FAO/WHO recommended standard for ideal proteins [10,11], indicating high nutritional value and functional potential.

Compared with conventional emulsions, nanoemulsions have garnered increasing academic attention in recent years due to their unique physicochemical properties, including a narrow particle size distribution, high transparency, superior thermodynamic stability, and enhanced bioavailability [12,13,14]. Current research predominantly employs small-molecule surfactants to construct nanoemulsion systems. These emulsifiers exhibit excellent surface activity and rapid adsorption kinetics, which significantly reduce the oil/water interfacial tension and yield nanoemulsions with smaller droplet sizes and more uniform distributions. However, from the perspective of adsorption–desorption equilibrium, small-molecule surfactants adsorbed at the interface possess relatively low adsorption energy, making them prone to desorption and thus compromising nanoemulsion stability [15]. Consequently, excessive surfactant concentrations are often required in practical applications to ensure system stability. Nevertheless, studies indicate that high intake of small-molecule surfactants may adversely affect human health, while the resulting nanoemulsions still exhibit limited stability [16]. To address these challenges, food-grade nanoemulsions have emerged as a promising alternative. Proteins, owing to their natural amphiphilicity, play a crucial role in food-grade emulsion systems. Notably, research has demonstrated that protein adsorption at oil/water interfaces is irreversible [17], a characteristic that provides unique advantages for constructing stable emulsion systems. Plant proteins, in particular, are widely utilized in nanoemulsion formulations due to their abundant availability, balanced nutritional profile, and rich essential amino acid content. For instance, Zafeer et al. demonstrated that zein-based nanoemulsions could effectively encapsulate thymol, enhancing its antibacterial and biofilm-inhibitory activity against multidrug-resistant *Campylobacter jejuni* [18]. Similarly, Awlqadr et al. employed chickpea protein isolate as an emulsifier to prepare nanoemulsions, achieving improved lutein encapsulation efficiency and prolonged system stability [19]. Additionally, Walia et al. reported that pea protein-stabilized nanoemulsions could effectively encapsulate vitamin D, both enhancing its bioaccessibility and providing a viable solution to vitamin D deficiency in elderly populations [20].

Nanoemulsion preparation methods can be classified into two categories based on energy input levels: high-energy emulsification and low-energy emulsification. Among these, high-energy emulsification relies on intense mechanical forces, with common equipment including high-pressure homogenizers and ultrasonic processors, which find wide application in the food industry for nanoemulsion production [21]. Ultrasonic homogenization, as a well-established nanoemulsion fabrication technique, achieves nanoscale transformation of emulsion systems through the physical effects induced by high-intensity ultrasound. The core mechanism of this technology lies in ultrasonication-induced cavitation. This process initially disrupts the oil/water interfacial tension via high-frequency vibrations to form a pre-emulsion. Subsequently, localized high-pressure shockwaves and microjets generated by cavitation further reduce droplet size, ultimately yielding a nanoemulsion system with uniform droplet size distribution [22]. Emulsions produced by ultrasonic homogenization exhibit long-term stability, while the equipment offers advantages such as ease of control, operational simplicity, and straightforward cleaning [23,24,25]. For instance, Zhu et al. employed ultrasonic emulsification with an ice bath temperature control system to prepare green cinnamon essential oil nanoemulsions, thereby effectively extending the postharvest shelf life of strawberries [26]. Similarly, Nie et al. developed black pepper essential oil nanoemulsions using this technique, demonstrating not only enhanced bioactivity of the essential oil but also exceptional storage stability, with consistent physicochemical properties maintained over a 30-day observation period [27].

Currently, there remains limited research on pepper seed protein isolate (PSPI) as a natural emulsifier for constructing nanoemulsion systems. In light of this, the present study first characterized the structural and functional properties of PSPI, followed by optimization of ultrasonic homogenization parameters for PSPI nanoemulsions using response surface methodology (RSM), with subsequent evaluation of their stability characteristics.

## 2. Materials and Methods

### 2.1. Materials and Chemicals

Pepper seeds were provided by Shujun Shuwei Chili Industry Development Co., Ltd. (Guizhou, China). Soy protein isolate (SPI) was purchased from Anyang Mantianxue Food Manufacturing Co., Ltd. (Anyang, China). Medium-chain triglycerides (MCTs) were sourced from Shanghai Yuanye Bio-Technology Co., Ltd. (Shanghai, China). All the other chemical reagents used were of analytical purity.

### 2.2. Preparation of PSPI

The PSPI was extracted following a modified protocol based on the method described by Manzoor et al. [11]. Briefly, 100 g of ground pepper seed powder was defatted by mixing with 1000 mL of *n*-hexane under continuous stirring for 4 h, followed by centrifugation and supernatant removal. This defatting procedure was repeated to ensure complete lipid extraction. The defatted powder was then mixed with distilled water at a 1:20 (*w*/*v*) ratio, and the pH was adjusted to 10.0 using 1 mol/L NaOH. After 2 h of continuous stirring at 25 °C, the mixture was centrifuged at 8000× *g* for 20 min at 4 °C and subsequently filtered. The resulting supernatant was acidified to pH 4.2 with 1 mol/L HCl to induce protein precipitation, followed by centrifugation under identical conditions. The precipitated protein was washed repeatedly with deionized water until a neutral pH was achieved. The collected protein was then redissolved and subjected to dialysis for 24 h at 4 °C. Finally, the purified protein solution was freeze-dried under vacuum to obtain PSPI. PSPI was extracted from 100 g of defatted chili seed powder, yielding 4.5 g after freeze-drying. The protein purity of the prepared PSPI was determined using the Kjeldahl method, yielding a purity of 92.37 ± 0.23%.

### 2.3. Amino Acid Composition

The amino acid composition of the sample was determined using acid hydrolysis. Precisely 100 mg of the sample was weighed into a pressure-resistant hydrolysis tube, followed by the addition of 10 mL of 6.0 mol/L hydrochloric acid (HCl) solution. High-purity nitrogen gas (≥99.99%) was purged into the tube for 30 s to eliminate residual air, after which the tube was immediately sealed. The hydrolysis tube was then placed in a constant-temperature drying oven and hydrolyzed at 110 ± 1 °C for 24 h. Upon completion of hydrolysis, the tube was allowed to cool to room temperature. The hydrolysate was filtered into a 50 mL volumetric flask and brought to volume with deionized water. A 2 mL aliquot of the filtrate was transferred to a 50 mL centrifuge tube and dried using a vacuum concentrator at 50 °C. The residue was reconstituted in 0.05 mol/L HCl and subsequently filtered through a 0.22 μm microporous membrane into an HPLC vial. The contents of 17 amino acids (excluding tryptophan) were quantified using an L-8900 amino acid analyzer (Hitachi High-Tech Corporation, Tokyo, Japan).

### 2.4. SDS-PAGE Electrophoresis

The protein solution (5 mg/mL) was prepared by mixing with SDS sample buffer containing β-mercaptoethanol (reducing conditions) or without β-mercaptoethanol (non-reducing conditions), followed by boiling for 5 min and cooling to room temperature. After centrifugation, the samples were stored at 4 °C for subsequent use. A 12% separating gel and a 5% stacking gel were prepared, and electrophoresis was performed using a DYCZ-24DN electrophoresis system (Beijing Liuyi Biotechnology Co., Ltd., Beijing, China). A 6 μL sample was loaded per well, with the initial voltage set at 80 V for 30 min, and then increased to 120 V. After electrophoresis, the gel was stained with Coomassie Brilliant Blue R-250 for 40 min and subsequently destained with a decolorization solution under gentle agitation (three times). Protein bands were visualized and imaged using a Molecular Imager Gel Doc XR+ system (Bio-Rad, Shanghai, China).

### 2.5. Fourier-Transform Infrared Spectroscopy (FTIR)

The PSPI sample was thoroughly mixed with potassium bromide (KBr) at a ratio of 1:100 (*w*/*w*) under a drying lamp and ground into a fine powder. The mixture was then pressed into a transparent pellet. The pellet was placed in a Nicolet iS20 FTIR spectrometer (Thermo Fisher Scientific, Waltham, MA, USA) with a pure KBr pellet used as the background reference. Spectra were recorded in the range of 400–4000 cm^−1^ at a resolution of 4 cm^−1^.

### 2.6. Determination of Functional Properties

#### 2.6.1. Determination of Water-Holding Capacity

The water-holding capacity (WHC) was determined as follows: A 10 mL empty centrifuge tube was weighed in advance. Then, 0.2 g of PSPI or SPI was dispersed in 20 mL of distilled water, with the pH adjusted to 3.0, 4.0, 5.0, 6.0, 7.0, or 8.0. A 5 mL aliquot of each pH-adjusted sample solution was transferred into the pre-weighed centrifuge tube and centrifuged at 3200× *g* for 10 min. The supernatant was discarded, and the tube was weighed again. The WHC was calculated as follows:

(1) $$\mathrm{WHC}=\frac{m_{2}-m_{1}}{m}\times 100$$

where *m* is the sample mass (g); *m*_1_ is the empty tube mass (g); and *m*_2_ is the tube mass after supernatant removal (g).

#### 2.6.2. Determination of Oil Holding Capacity

The oil-holding capacity (OHC) was determined with slight modifications based on the method described by Jeong [28]. Briefly, a centrifuge tube was weighed to record its empty mass (*m*_1_). Then, 0.5 g of the PSPI (*m*) was mixed with 5 mL of salad oil by vortexing to ensure homogeneity. The mixture was centrifuged at 5000× *g* for 30 min. After centrifugation, the upper layer of salad oil was carefully discarded, and the tube was weighed again (*m*_2_). The OHC was calculated using the following equation:

(2) $$\mathrm{OHC}=\frac{m_{2}-m_{1}}{m}\times 100$$

### 2.7. Interfacial Tension Measurement

The interfacial tension between medium-chain triglycerides (MCTs) and water was measured using a Theta Lite optical tensiometer (Biolin Scientific, Stockholm, Sweden). Aqueous solutions of PSPI and SPI at varying concentrations (0.1%, 0.3%, and 0.5%) were tested, with ultrapure water serving as the blank control. For measurement, a high-precision syringe was used to immerse the pipette tip into MCT, followed by dispensing a single droplet of the sample solution. The instrument continuously monitored the dynamic shape changes in the droplet, and the interfacial tension was automatically calculated based on the Young–Laplace equation.

### 2.8. Ultrasonic Emulsifying Process for PSPI Nanoemulsions

The nanoemulsions were prepared using an ultrasonic emulsification method. PSPI was dissolved in distilled water at varying concentrations to form the aqueous phase, while MCT served as the oil phase. The two phases were mixed at a ratio of 95:5 (*v*/*v*) and pre-homogenized using an IKA Ultra-Turrax T18 high-speed homogenizer (Sykam, Germany) at 15,000 rpm for 2 min to obtain a coarse emulsion. The coarse emulsion was then subjected to ultrasonication using a Scientz JY99-IIDN ultrasonic cell disruptor (Ningbo Scientz Biotechnology Co., Ltd., Ningbo, China) under ice bath conditions. The ultrasonication parameters were set with a pulse mode of 2 s on and 2 s off to prevent overheating, while maintaining the sample temperature below 25 °C throughout the process. The resulting nanoemulsion was characterized for droplet size and polydispersity index (PDI) using a Malvern Nano ZS laser particle size analyzer (Malvern Instruments, Worcestershire, UK). Prior to measurement, the emulsion was diluted 1000-fold (*v*/*v*) with distilled water and equilibrated at 25 °C for 2 min to minimize multiple light scattering effects.

### 2.9. Single-Factor Optimization of Ultrasonic Homogenization Parameters

The influence of protein concentration (0.1–0.8%), ultrasonic power (200–700 W), and ultrasonic time (60–480 s) on the droplet size and polydispersity index (PDI) of nanoemulsions was systematically investigated using ice bath temperature control. Nanoemulsion droplet size and PDI served as the primary evaluation indices throughout this study.

### 2.10. Optimization of Ultrasonic Homogenization Using Response Surface Methodology

Based on the single-factor experimental results, response surface methodology (RSM) was employed to optimize the ultrasonic homogenization process for nanoemulsion preparation. The optimization parameters included ultrasonic power (X_1_), protein concentration (X_2_), and ultrasonic time (X_3_), with the particle size (R, nm) of the nanoemulsion serving as the response variable. This experimental design allowed for systematic evaluation of the parameter interactions and determination of optimal processing conditions to achieve minimal particle size.

### 2.11. Stability of PSPI Nanoemulsions

#### 2.11.1. Effect of pH

The pH of PSPI nanoemulsions was adjusted to 3.0, 4.0, 5.0, 6.0, 7.0, 8.0, and 9.0 using 1.0 mol/L HCl and NaOH solutions. After standing at room temperature for 30 min, the visual appearance of the nanoemulsions was recorded, and the samples were collected to determine changes in droplet size under different pH conditions.

#### 2.11.2. Effect of Ionic Strength

To 10 mL of PSPI nanoemulsion, NaCl solution (1.0 mol/L) was added to adjust the Na^+^ concentration to 0, 20, 40, 60, 80, or 100 mmol/L. Ultrapure water was then added to equalize the total volume of all the samples. The nanoemulsions were kept at room temperature for 30 min, followed by visual observation and droplet size measurement at different ionic strengths.

#### 2.11.3. Effect of Temperature

PSPI nanoemulsions were incubated in water baths at different temperatures (40, 50, 60, 70, 80, 90, and 100 °C) for 20 min, and then rapidly cooled to room temperature. The visual stability was assessed, and the droplet size was measured after thermal treatment.

### 2.12. Statistical Analysis

All the experiments were performed in triplicate, with the results expressed as mean ± standard deviation. Significant differences among treatments were analyzed using the IBM SPSS Statistics 21.0 software with Duncan’s multiple range test (*p* < 0.05). Data visualization was performed using the Origin 2024 software, while response surface methodology (RSM) design and analysis were conducted using Design-Expert 13.0 software.

## 3. Results and Discussion

### 3.1. Amino Acid Composition Analysis of PSPI

The nutritional value of proteins primarily depends on the types, quantities, and composition ratios of essential amino acids they contain. As an important plant protein source, PSPI demonstrates significant advantages in meeting food industry demands and promoting sustainable nutritional development. As shown in Table 1, PSPI contains 17 amino acids (tryptophan was not detected due to destruction during acid hydrolysis). Consistent with Embaby et al. [29] yet distinct from Yılmaz et al. [30] (who reported no cystine), this discrepancy may reflect methodological differences in analysis. Notably, essential amino acids accounted for 39.35 ± 0.06% of the total amino acid content, closely approaching the FAO/WHO-recommended standard (40%) for ideal proteins. The amino acid profile revealed PSPI’s unique nutritional and functional characteristics. The PSPI hydrophilic surface residues (Asp/Glu) have a high content, which increases the negative charge on the protein surface and enhances the emulsifying property. Liao et al. significantly increased the Asp/Glu content of gluten protein through deamidation modification, thereby increasing the surface negative charge and improving the emulsifying activity index [31]. Glutamic acid (14.11 ± 0.02 g/100 g), the most abundant amino acid in PSPI, not only functions as a neurotransmitter but has also been recently implicated in tumor regulation [32]. Furthermore, PSPI is rich in glycine (9.96 ± 0.03 g/100 g) and leucine (9.68 ± 0.00 g/100 g). Glycine has demonstrated significant value in preventing and managing metabolic diseases such as cardiovascular disorders and diabetes [33], while leucine plays crucial roles in protein synthesis, energy metabolism, and cellular signaling. Unlike conventional cereal proteins (e.g., wheat or corn), PSPI’s first limiting amino acid is not lysine (Lys) but rather cysteine (Cys). This distinctive composition enables PSPI to effectively compensate for amino acid deficiencies in cereal proteins, offering new opportunities for developing functional foods with balanced amino acid profiles. Plant proteins containing these amino acids holistically support human health through multiple pathways: facilitating muscle growth and recovery, boosting immune function, and delivering antioxidant/anti-aging properties, thereby serving as an excellent nutritional option.

### 3.2. SDS-PAGE Electrophoresis Analysis

**Figure 1:**
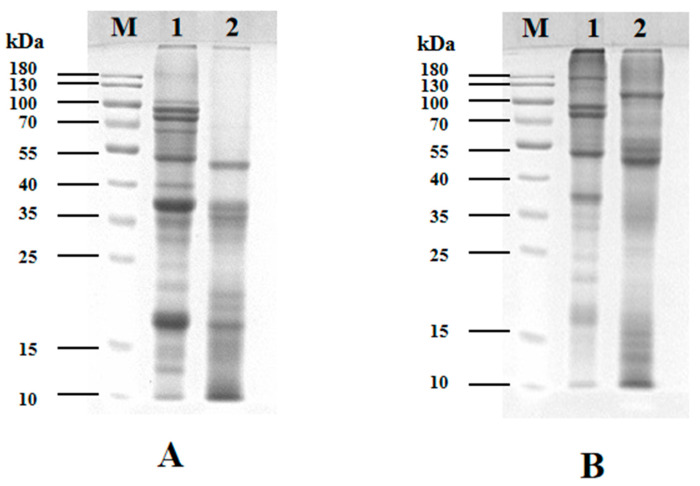
SDS-PAGE electrophoretic profiles of SPI and PSPI under (**A**) reducing and (**B**) non-reducing conditions (M: molecular weight markers).

Figure 1 presents the SDS-PAGE electrophoretic profiles of SPI and PSPI. Under reducing conditions (with β-mercaptoethanol), significant differences were observed in subunit composition and molecular weight distribution between the two proteins (Figure 1A). SPI (lane A1) exhibited a broad band distribution (10–100 kDa), showing characteristic bands at 70 kDa (7S α’ subunit), 35–40 kDa (11S acidic subunits), and 15–25 kDa (11S basic subunits). In contrast, PSPI (lane A2) displayed bands primarily distributed between 10 and 55 kDa, with prominent bands observed at 55 kDa, 35–40 kDa, and 15–25 kDa. This distinct electrophoretic pattern likely reflects functional differences between the proteins: while SPI primarily serves storage functions, PSPI may be enriched with small molecular-weight defense-related proteins [34].

To investigate subunit interactions, both proteins were analyzed by SDS-PAGE under non-reducing conditions (without β-mercaptoethanol), as shown in Figure 1B. Compared to the reducing condition, SPI (lane 1) developed new bands in the 130–180 kDa range, accompanied by reduced band intensity at 35–40 kDa and 15–25 kDa regions, with complete disappearance of bands below 15 kDa. Similarly, PSPI under non-reducing conditions (lane 2) showed new high-molecular-weight bands (100–300 kDa), enhanced intensity at 55 kDa, and disappearance of bands in the 35–40 kDa and 15–25 kDa regions. These observations demonstrate that subunits in both proteins form high-molecular-weight complexes through intermolecular disulfide bonds.

### 3.3. FT-IR Analysis

**Figure 2:**
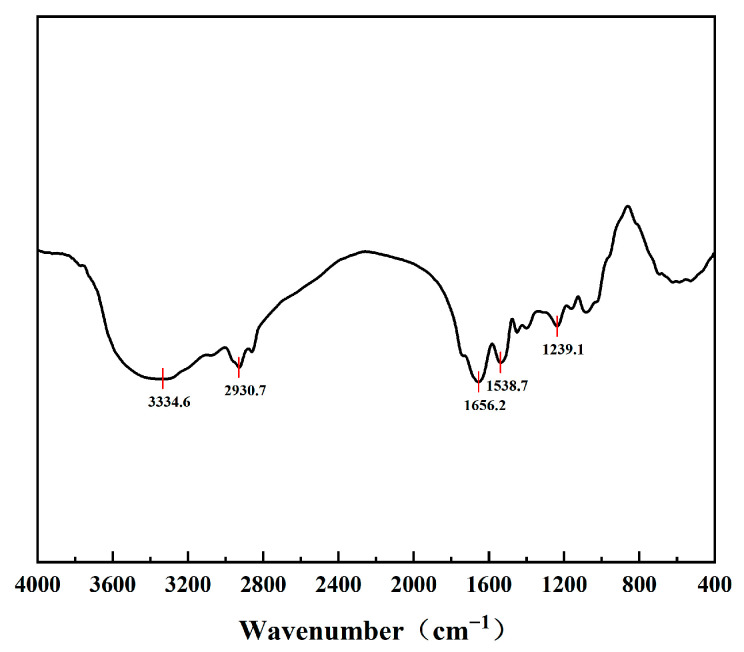
FT-IR spectra of PSPI.

Fourier-Transform Infrared Spectroscopy (FT-IR) is a commonly used technique for characterizing the secondary structure of proteins and their microenvironmental characteristics. The FT-IR spectrum of PSPI (Figure 2) was observed to exhibit the following characteristic absorption peaks: a strong absorption peak at 1656.2 cm^−1^, which was attributed to the amide I band (mainly C=O stretching vibration), and was suggested to potentially represent α-helix as the main secondary structure of PSPI; the amide II band at 1538.7 cm^−1^ (N-H bending coupled with C-N stretching) was found to be consistent with this attribution [35]. A broad peak at 3334.6 cm^−1^ was derived from N-H and O-H stretching vibrations, and was reflected in the hydrogen bond network between the protein and water molecules [36]. The absorption peak at 2930.7 cm^−1^ corresponded to the stretching vibration of the aliphatic C-H group, which was derived from the amino acid side chains. The absorption peak at 1239.1 cm^−1^ of the amide III region was suggested to possibly represent β-sheet or other conformational components [37].

### 3.4. Functional Properties

#### 3.4.1. Water-Holding Capacity (WHC) Analysis

**Figure 3:**
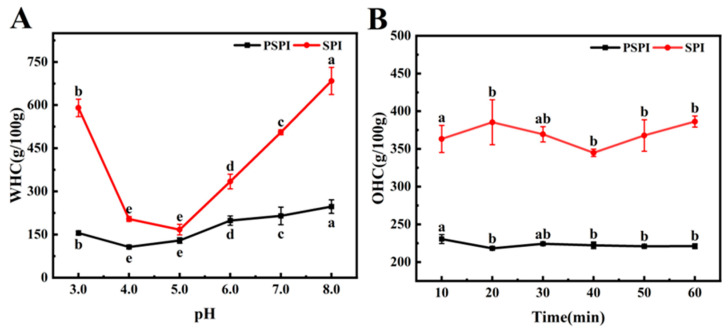
Water-holding (**A**) and oil-holding (**B**) capacities of PSPI and SPI.(The different letters in the figure indicate that there are significant differences among the data, *p* < 0.05).

The water-holding capacity (WHC) of proteins reflects their ability to bind and retain water under centrifugal conditions [28]. This property primarily depends on the molecular characteristics of proteins, including amino acid composition, net charge, spatial conformation, and the distribution of hydrophilic/hydrophobic groups, while also being influenced by environmental factors such as temperature, pH, and ionic strength [38]. As shown in Figure 3A, soy protein isolate (SPI) exhibited superior WHC compared to PSPI across all the tested pH conditions. With increasing pH, both proteins initially showed a decrease followed by an increase in WHC. SPI reached its lowest WHC at pH 5.0, whereas PSPI exhibited its minimum at pH 4.0, a phenomenon closely related to their respective isoelectric points. As the pH deviated from the isoelectric point, the WHC of both proteins increased, likely due to pH-induced conformational changes that exposed additional water-binding sites, enhanced protein polar charges, and promoted the proportion of water-binding proteins [39]. This finding aligns with the study by Tang et al. on the effects of pH on the functional properties of plant and dairy proteins, where they similarly observed enhanced water-holding capacity as proteins moved away from their isoelectric points [40].

#### 3.4.2. Oil-Holding Capacity Analysis

The oil-holding capacity (OHC) of proteins is a critical functional property that reflects their ability to bind and retain lipids, representing another form of emulsification. In food processing, OHC not only plays a vital role in maintaining product stability but also significantly improves the retention of flavor compounds [39]. The effects of resting time on OHC were evaluated for PSPI and SPI. As illustrated in Figure 3B, SPI demonstrated superior OHC compared to PSPI, and prolonged resting time had no significant impact on the OHC of either protein. This suggests that the lipid-binding capacity of both proteins reached saturation shortly after exposure, resulting in negligible changes with extended resting time. According to Shen et al. [41], the OHC of proteins is influenced by their concentration, surface area, lipophilicity, and the number of exposed hydrophobic amino acids.

### 3.5. Interfacial Tension Analysis

**Figure 4:**
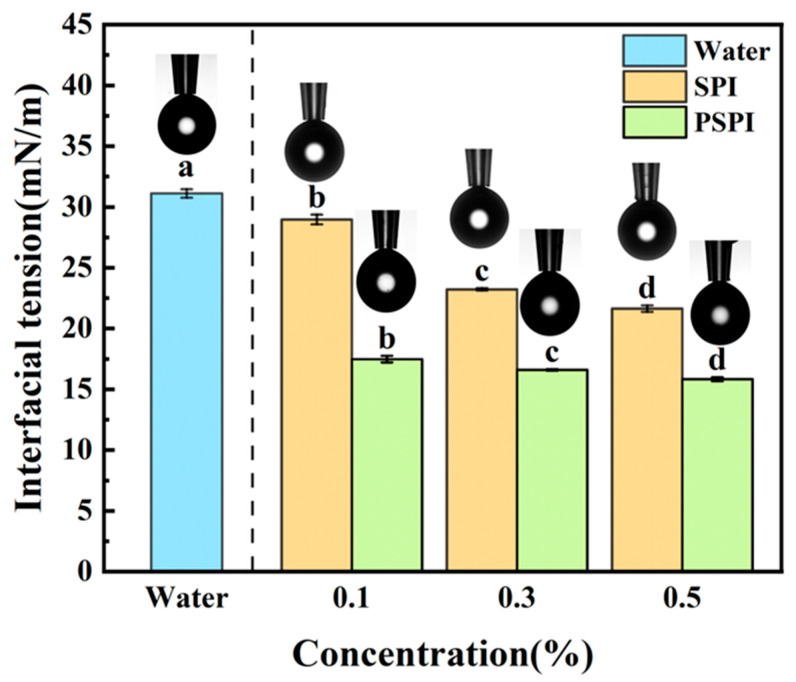
Effect of PSPI and SPI on MCT/water interfacial tension (schematic representations of pendant drop morphology at respective concentrations are displayed above the bar graph, the different letters in the figure indicate that there are significant differences among the data, *p* < 0.05).

Interfacial tension, a key physical parameter for evaluating the stability of emulsion systems, reflects the thermodynamic properties of air/water and oil/water interfaces. Both proteins and small-molecule surfactants can adsorb at these interfaces, reducing the system’s interfacial free energy [42]. Pendant drop tensiometry is widely used for measuring interfacial properties due to its operational simplicity and broad applicability, providing critical insights into intermolecular interactions and molecular aggregation behavior [43]. In this study, the effects of different concentrations of PSPI and SPI solutions on MCT/water interfacial tension were investigated. As shown in Figure 4, both SPI and PSPI significantly reduce interfacial tension compared to the blank (31.11 ± 0.35 mN/m) (*p* < 0.05), with the reduction effect becoming more pronounced at higher protein concentrations. At a protein concentration of 0.5%, SPI reduced interfacial tension to 21.65 ± 0.27 mN/m, while PSPI achieved a greater reduction to 15.84 ± 0.16 mN/m. Under identical protein concentrations, PSPI exhibited superior efficiency in lowering interfacial tension compared to SPI, indicating stronger interfacial adsorption capacity and better emulsifying performance.

### 3.6. Analysis of Factors Influencing Ultrasonic Homogenization

#### 3.6.1. Ultrasonic Power

**Figure 5:**
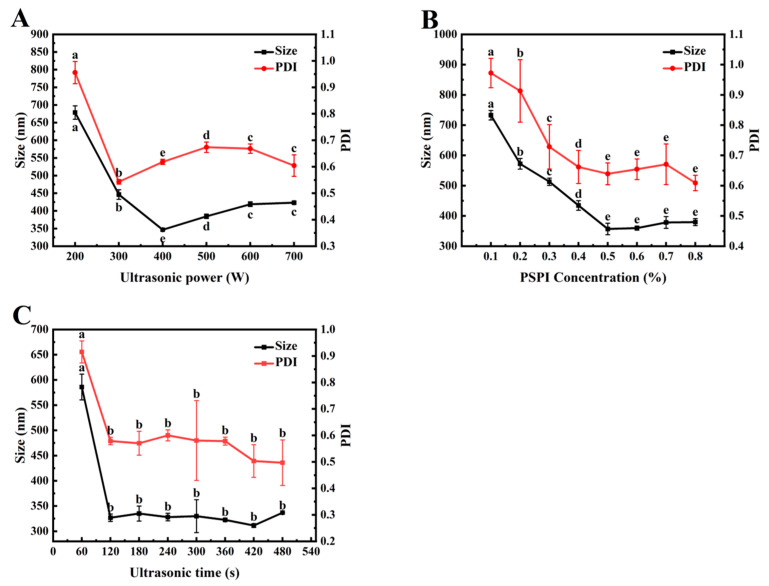
Effects of ultrasonic power (**A**), PSPI concentration (**B**), and ultrasonic time (**C**) on the median droplet size and PDI of PSPI nanoemulsion (The different letters in the figure indicate that there are significant differences among the data, *p* < 0.05).

Ultrasonic treatment can disrupt larger oil droplets and promote the rapid formation of homogeneous nanoemulsions [44]. As shown in Figure 5A, the droplet size and polydispersity index (PDI) of the emulsion exhibit a significant decreasing trend (*p* < 0.05) within the ultrasonic power range of 200–400 W. This phenomenon may be attributed to the exposure of hydrophobic groups at the interface during ultrasonication, which enhances protein adsorption at the oil/water (O/W) interface, significantly reduces interfacial tension, and thereby improves emulsion stability. Notably, when the ultrasonic power was increased to 500–700 W, the emulsion droplet size gradually increased. This may be due to excessive cavitation bubble density under high-power conditions, leading to localized energy accumulation and subsequent droplet re-aggregation under intense shear forces [45]. A similar observation was reported by Yang et al. in their study on the size refinement mechanism of metal nanoparticles under ultrasonication, where further increases in ultrasonic power did not enhance particle refinement but increased particle sizes [46]. In this study, the nanoemulsion prepared at 400 W exhibited the smallest droplet size and PDI, indicating uniform and finely dispersed droplets. Therefore, the ultrasonic power range of 300–500 W was selected for subsequent optimization experiments.

#### 3.6.2. PSPI Concentration

Proteins stabilize emulsions by forming a viscoelastic interfacial film, whose integrity is reinforced by electrostatic repulsion, steric hindrance, and a three-dimensional continuous-phase network [47]. The stability of emulsions depends not only on the type of protein but also on the protein’s chemical structure, molecular weight, charge density, aggregation state, and concentration in the system [48]. As illustrated in Figure 5B, increasing protein concentration gradually decreased emulsion droplet size and PDI, eventually reaching a plateau. This suggested that higher protein concentrations increased interfacial coverage, inhibiting droplet aggregation and resulting in smaller emulsion particles. Similarly, Ren et al. reported that increasing the concentration of Moringa seed protein (0.125–0.1%) reduced emulsion droplet size [49]. In this study, a PSPI concentration of 0.5% yields a low PDI, uniform droplet size distribution, and good emulsion dispersibility [50]. Based on these findings, a protein concentration range of 0.4–0.6% was selected for further optimization to balance emulsion stability with cost efficiency.

#### 3.6.3. Ultrasonic Time

Figure 5C demonstrates the effect of ultrasonic treatment time on nanoemulsion droplet size. Prolonged ultrasonication initially reduced both droplet size and PDI, followed by stabilization. After 120 s, the average particle size reached its minimum (327.17 ± 14.36 nm) and remained stable. This was because ultrasonic cavitation broke down droplets into smaller droplets while promoting rapid PSPI adsorption at the oil/water interface, enhancing emulsification. As the droplet size decreased, higher energy density was required for further fragmentation. When the input energy reached a dynamic equilibrium with the droplet breakup threshold, the droplet size distribution stabilized. Pratap et al. also observed that beyond an optimal ultrasonication time (T_opt_), extended treatment had negligible effects on droplet size reduction. This suggests a limit to the ultrasonication effect beyond which further size reduction is insignificant [51]. Considering energy efficiency and emulsification performance, an ultrasonication time range of 60–180 s was selected for subsequent optimization experiments.

### 3.7. Analysis of Response Surface Optimization Results

The experimental design and results of response surface methodology are presented in Table 2. Based on the results from single-factor experiments, the variation ranges of influencing factors were determined. A response surface optimization experiment was designed considering three factors: ultrasonic power (X_1_), PSPI concentration (X_2_), and ultrasonication time (X_3_), with the average droplet size (R, nm) of the nanoemulsion as the response value. The quadratic polynomial regression model was established as follows:

R = 385.32 − 118.37X_1_
+ 22.20X_2_ − 150.79X_3_ − 46.23X_1_X_2_
+ 92.03X_1_X_3_ − 20.86X_2_X_3_ + 64.94X_1_^2^
+ 9.82X_2_^2^ + 133.50X_3_^2^

Regression and variance analyses revealed that the model for nanoparticle size was highly significant (*p* < 0.01) with a non-significant lack-of-fit term (*p* > 0.05). The coefficient of determination R^2^ = 0.9968 indicated excellent agreement between experimental data and model predictions, demonstrating the model’s capability to accurately reflect the variation pattern of nanoparticle size [52]. The adjusted coefficient of determination R^2^_Adj_ = 0.9926 suggested that approximately 99.26% of the variation in droplet size response values could be explained by this model. Therefore, this quadratic polynomial regression model could be effectively used to analyze and predict the actual particle size of nanoemulsions. An F-value analysis showed that the influencing factors followed this order of significance: ultrasonic time > ultrasonic power > PSPI concentration.

**Figure 6:**
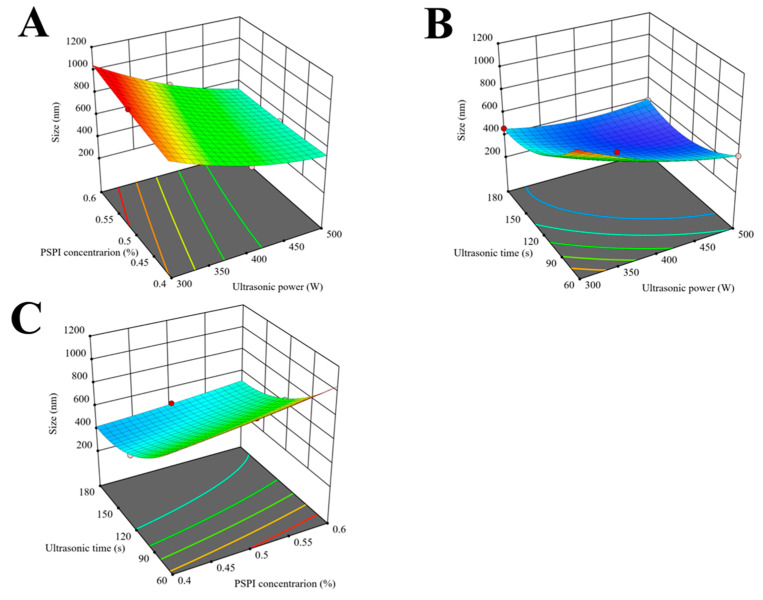
Response surface plots for the ultrasonic homogenization of PSPI nanoemulsion. ((**A**): three-dimensional plot of the interactive effects of PSPI concentration and Ultrasonic power on canoemulsion particle Size; (**B**): three-dimensional plot of the interactive effects of Ultrasonic time and Ultrasonic power on nanoemulsion particle size; (**C**): three-dimensional plot of the interactive effects of Ultrasonic time and PSPI concentration on nanoemulsion particle size).

The response surface analysis of interaction effects between paired factors is illustrated in Figure 6. Ultrasonic time exhibited a superior influence on nanoemulsion droplet size compared to ultrasonic power and PSPI concentration. Prolonged ultrasonic time allowed more effective utilization of cavitation effects, thereby more efficiently reducing droplet size. This observation was consistent with the findings of Saba et al. in their study of ultrasound parameters on the physical characteristics of lycopene emulsions, where they also found that ultrasonic time predominantly controlled nanoparticle size at relatively low ultrasonic power levels [45].

Through RSM analysis of the regression model, the optimal process parameters were determined as follows: PSPI concentration 0.53%, ultrasonic power 490.66 W, and ultrasonic time 129.53 s. For practical operation, these parameters were adjusted to the following: PSPI concentration 0.53%, ultrasonic power 500 W, and ultrasonic time 130 s. Triplicate verification experiments yielded an average particle size of 319 ± 2 nm for PSPI nanoemulsions, closely matching the predicted value of 318 nm. This excellent agreement between response surface model predictions and actual measurements confirmed the reliability of the regression model.

### 3.8. Stability of PSPI Nanoemulsion

#### 3.8.1. pH

**Figure 7:**
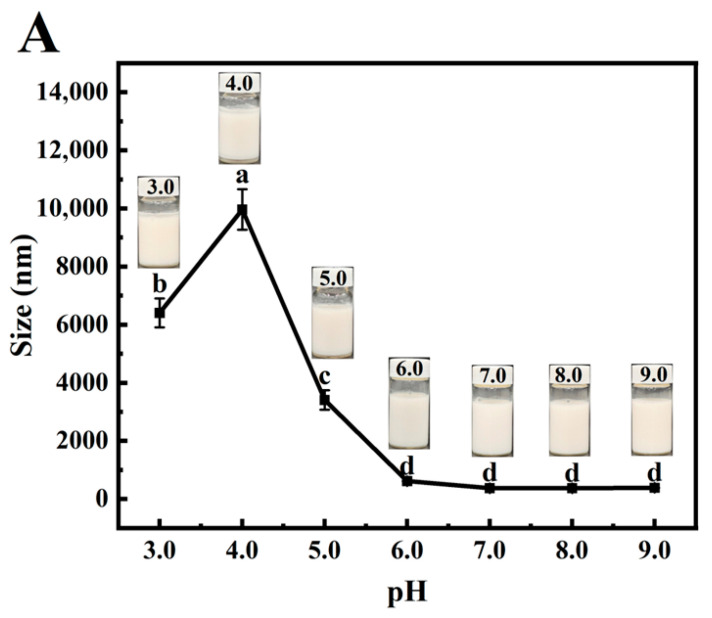

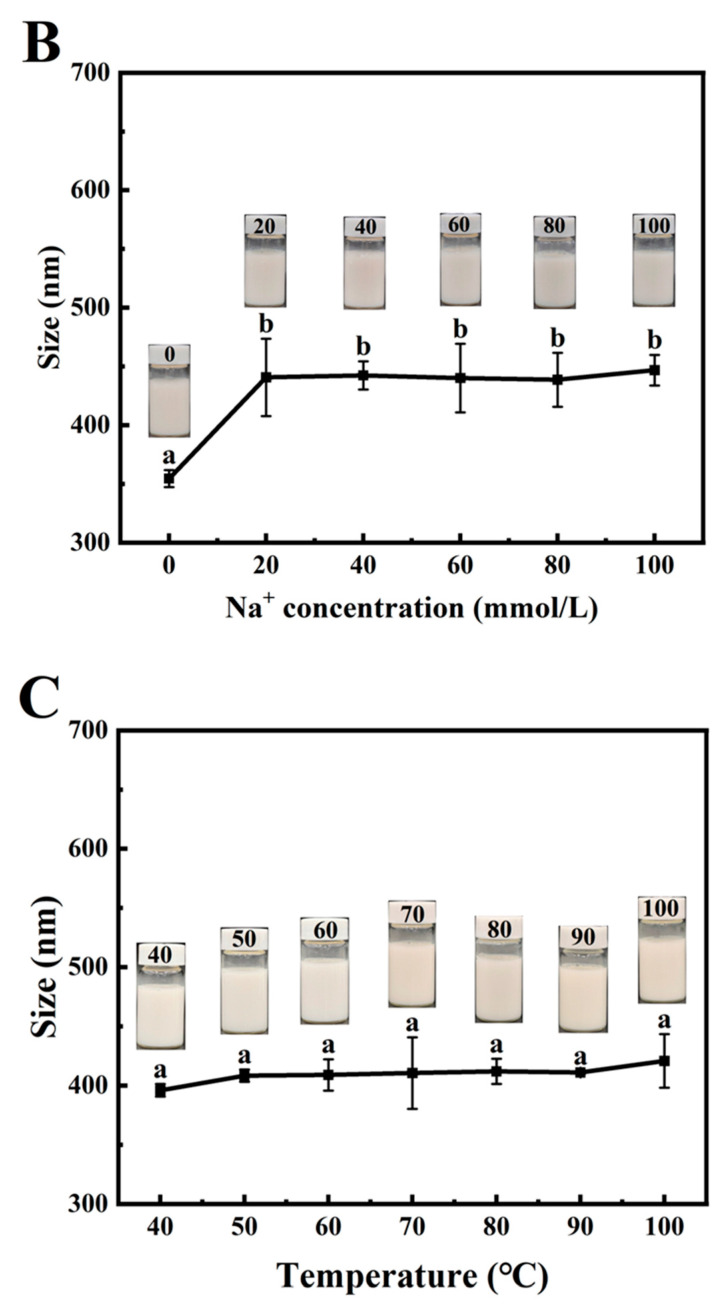
Effects of pH (**A**), Na^+^ concentration (**B**), and temperature (**C**) on the particle size of PSPI-stabilized nanoemulsions (the image above the line graph shows the emulsion appearance under these conditions, the different letters in the figure indicate that there are significant differences among the data, *p* < 0.05).

Emulsion instability manifests through flocculation, aggregation, creaming, coalescence, and Ostwald ripening, with pH being a critical factor affecting emulsion flocculation [53]. Surface-active proteins can stabilize emulsions at relatively low concentrations, but the resulting droplets exhibit high sensitivity to pH variations [54]. Figure 7A presents the visual appearance and droplet size changes in PSPI-stabilized emulsions at different pH levels. Phase separation was observed within 30 min of storage at pH 3–5, with the most pronounced instability occurring at pH 4. This phenomenon likely results from reduced protein solubility and hydration capacity near the isoelectric point, leading to diminished electrostatic repulsion [39]. In contrast, emulsions maintained a stable appearance and consistent particle size at pH 7–9, suggesting that balanced electrostatic repulsion enhances emulsion stability in this pH range.

#### 3.8.2. Ionic Strength

Ionic stability represents a crucial parameter for evaluating emulsion characteristics. Salt addition may alter protein conformation and screen electrostatic repulsion between protein-coated oil droplets [55]. As shown in Figure 7B, the PSPI nanoemulsion maintained an average particle size of approximately 354 nm without Na^+^ addition. Particle size increased significantly with Na^+^ concentration elevation from 0 to 20 mmol/L, stabilizing around 441 nm at 40–100 mmol/L. This size enlargement reflects reduced electrostatic repulsion between droplets upon salt addition. Similar trends have been reported for emulsions stabilized by flaxseed gum [56] and β-conglycinin [57]. Remarkably, all the samples maintained a homogeneous appearance throughout the ionic strength gradient without phase separation or flocculation, demonstrating excellent ionic stability of PSPI nanoemulsions. This property ensures their potential application in complex food systems.

#### 3.8.3. Thermal Stability

Figure 7C reveals that PSPI nanoemulsions maintained a consistent droplet size across temperature variations. Although 100 °C treatment caused a slight size increase to approximately 420 nm, the system remained stable. This behavior suggested that thermal denaturation of PSPI exposes hydrophobic regions while maintaining emulsifying capacity. These findings align with Ma et al., who reported superior thermal stability in emulsions prepared with low-concentration soy protein [58]. The absence of visible changes in emulsion appearance during thermal treatment confirms the strong heat resistance of PSPI-stabilized nanoemulsions, supporting their potential incorporation into thermally processed food emulsion systems. The combined pH, ionic, and thermal stability results position PSPI nanoemulsions as promising candidates for diverse food applications requiring robust physicochemical stability.

## 4. Conclusions

The PSPI demonstrated a rich amino acid profile comprising 17 different amino acids, with glutamic acid being the most abundant. The SDS-PAGE analysis revealed that PSPI subunits were primarily distributed between 10 and 55 kDa, with some subunits interconnected via disulfide bonds. While PSPI exhibited superior interfacial tension reduction capability compared to SPI, its water-holding and oil-holding capacities were comparatively lower. The ultrasonic emulsifying method proved effective for preparing PSPI-stabilized nanoemulsions, with PSPI concentration, ultrasonication time, and power significantly influencing emulsion droplet size. -Through response surface methodology optimization, the optimal parameters within the experimental range were determined as follows: PSPI concentration 0.53%, ultrasonica time 130 s, and ultrasonic power 500 W. Under these optimized conditions, the resulting nanoemulsions achieved a droplet size of 319 ± 2 nm, closely matching the predicted value of 318 nm. The prepared nanoemulsion exhibited extremely high stability under conditions of medium alkalinity, variations in temperature, and ionic strength. These findings significantly enhance the practical value of PSPI while providing crucial technical references for developing innovative nanoemulsion systems in food industry applications.

## Figures and Tables

**Table 1:** Amino acid composition of PSPI (percentage of total detected amino acids).

NEAA	Content (g/100 g)	EAA	Content (g/100 g)
Asp	9.26 ± 0.01	Thr	4.79 ± 0.01
Ser	5.51 ± 0.03	Val	5.11 ± 0.01
Glu	14.11 ± 0.02	Met	1.75 ± 0.03
Gly	9.96 ± 0.03	Ile	5.05 ± 0.01
Ala	8.00 ± 0.02	Leu	9.68 ± 0.00
Cys	0.81 ± 0.01	Phe	5.23 ± 0.01
Tyr	2.70 ± 0.01	Lys	5.26 ± 0.01
His	2.50 ± 0.01		
Arg	9.18 ± 0.01		
Pro	1.16 ± 0.01		
Total NEAA	60.65 ± 0.06	Total EAA	39.35 ± 0.06

**Table 2:** Experimental design and RSM results.

RSM Experiment					ANOVA					
Run	X_1_ (W)	X_2_ (%)	X_3_ (s)	R (nm)	Source	Sum of Squares	df	Mean Square	F-Value	*p*-Value
1	400 (0)	0.4 (−1)	60 (−1)	626.47 ± 6.61	Model	440,800	9	48,982.64	240.40	<0.0001
2	300 (−1)	0.4 (−1)	120 (0)	506.20 ± 2.71	X_1_	112,100	1	112,100	550.11	<0.0001
3	400 (0)	0.4 (−1)	180 (1)	376.90 ± 6.10	X_2_	3942.72	1	3942.72	19.35	0.0032
4	400 (0)	0.6 (1)	60 (−1)	722.10 ± 4.84	X_3_	181,900	1	181,900	892.78	<0.0001
5	400 (0)	0.6 (1)	180 (1)	389.10 ± 12.15	X_1_X_2_	8547.00	1	8547.00	41.95	0.0003
6	500 (1)	0.5 (0)	180 (1)	392.90 ± 5.98	X_1_X_3_	33,874.40	1	1740.28	166.25	<0.0001
7	500 (1)	0.5 (0)	60 (−1)	520.73 ± 10.78	X_2_X_3_	1740.28	1	17,754.37	8.54	0.0223
8	300 (−1)	0.5 (0)	60 (−1)	958.67 ± 33.24	X_1_^2^	17,754.37	1	405.96	87.14	<0.0001
9	500 (1)	0.6 (1)	120 (0)	321.50 ± 4.28	X_2_^2^	405.96	1	75,043.86	1.99	0.2010
10	400 (0)	0.5 (0)	120 (0)	381.17 ± 7.00	X_3_^2^	75,043.86	1	203.75	368.31	<0.0001
11	400 (0)	0.5 (0)	120 (0)	380.43 ± 3.50	Residual	1426.26	7	327.19		
12	400 (0)	0.5 (0)	120 (0)	403.20 ± 1.63	Lack of fit	981.56	3		2.94	0.1621
13	300 (−1)	0.5 (0)	180 (1)	462.73 ± 10.07	Credibility analysis of the regression equations					
14	300 (−1)	0.6 (1)	120 (0)	633.53 ± 2.60	Std. dev	14.27	R-squared	0.9968		
15	400 (0)	0.5 (0)	120 (0)	385.63 ± 5.00	Mean	483.32	Adj. R-squared	0.9926		
16	500 (0)	0.4 (−1)	120 (0)	379.07 ± 3.88	Second-order polynomial equation					
17	400 (0)	0.5 (0)	120 (0)	376.167 ± 9.22	R = 385.32 − 118.37X_1_ + 22.20X_2_ − 150.79X_3_ − 46.23X_1_X_2_ + 92.03 X_1_X_3_ − 20.86X_2_X_3_ + 64.94X_1_^2^ + 9.82X_2_^2^ + 133.50X_3_^2^					

## Data Availability

The original contributions presented in this study are included in the article. Further inquiries can be directed to the corresponding author(s).

## References

[1] Wang F., Ma Y., Wang Y., Zhao L., Liao X. (2021). Physicochemical properties of seed protein isolates extracted from pepper meal by pressure-assisted and conventional solvent defatting. Food Funct..

[2] Hsu C.L., Yen G.C. (2007). Effects of capsaicin on induction of apoptosis and inhibition of adipogenesis in 3T3-L1 cells. J. Agric. Food Chem..

[3] Leung F.W. (2008). Capsaicin-sensitive intestinal mucosal afferent mechanism and body fat distribution. Life Sci..

[4] Malagarie-Cazenave S., Olea-Herrero N., Vara D., Díaz-Laviada I. (2009). Capsaicin, a component of red peppers, induces expression of androgen receptor via PI3K and MAPK pathways in prostate LNCaP cells. FEBS Lett..

[5] Sung J., Lee J. (2016). Capsicoside G, a furostanol saponin from pepper (*Capsicum annuum* L.) seeds, suppresses adipogenesis through activation of AMP-activated protein kinase in 3T3-L1 cells. J. Funct. Foods.

[6] Sim K.H., Sil H.Y. (2008). Antioxidant activities of red pepper (*Capsicum annuum*) pericarp and seed extracts. Int. J. Food Sci. Technol..

[7] Azlan A., Sultana S., Huei C.S., Razman M.R. (2022). Antioxidant, anti-obesity, nutritional and other beneficial effects of different chili pepper: A review. Molecules.

[8] Chouaibi M., Rezig L., Hamdi S., Ferrari G. (2019). Chemical characteristics and compositions of red pepper seed oils extracted by different methods. Ind. Crops Prod..

[9] Cvetković T., Ranilović J., Jokić S. (2022). Quality of pepper seed by-products: A review. Foods.

[10] Firatligil-Durmus E., Evranuz O. (2010). Response surface methodology for protein extraction optimization of red pepper seed (Capsicum frutescens). LWT-Food Sci. Technol..

[11] Manzoor M.F., Waseem M., Diana T., Wang R., Ahmed Z., Ahmed I.A.M., Ali M., An-Zeng X. (2025). Ultrasound-assisted modification to improve the red pepper seed protein isolate structural, functional, and antioxidant properties. Int. J. Biol. Macromol..

[12] Silva H.D., Cerqueira M.Â., Vicente A.A. (2012). Nanoemulsions for food applications: Development and characterization. Food Bioprocess Technol..

[13] Wang T., Luo Y. (2019). Biological fate of ingested lipid-based nanoparticles: Current understanding and future directions. Nanoscale.

[14] Zhao T., Yan X., Sun L., Yang T., Hu X., He Z., Liu F., Liu X. (2019). Research progress on extraction, biological activities and delivery systems of natural astaxanthin. Trends Food Sci. Technol..

[15] Karthik P., Ezhilarasi P.N., Anandharamakrishnan C. (2017). Challenges associated in stability of food grade nanoemulsions. Crit. Rev. Food Sci. Nutr..

[16] Johnson P., Trybala A., Starov V., Pinfield V.J. (2021). Effect of synthetic surfactants on the environment and the potential for substitution by biosurfactants. Adv. Colloid Interface Sci..

[17] Dickinson E. (1999). Adsorbed protein layers at fluid interfaces: Interactions, structure and surface rheology. Colloids Surf. B Biointerfaces.

[18] Zafer N., Imran M. (2024). Impact of phytoceuticals: Thymol-loaded zein-based nano-antimicrobials to combat resistant zoonotic pathogen. Ind. Crops Prod..

[19] Awlqadr F.H., Ghanbarzadeh B., Altemimi A.B., Arab K., Dadashi S., Pezeshki A., Hesarinejad M.A., Abedelmaksoud T.G. (2025). Encapsulation of lutein in nanoemulsions: Comparative evaluation of chickpea and soy protein isolates on physicochemical stability, antioxidant activity, and rheological properties. Food Chem. X.

[20] Walia N., Chen L. (2020). Pea protein based vitamin D nanoemulsions: Fabrication, stability and in vitro study using Caco-2 cells. Food Chem..

[21] McClements D.J. (2011). Edible nanoemulsions: Fabrication, properties, and functional performance. Soft Matter.

[22] Stepišnik Perdih T., Zupanc M., Dular M. (2019). Revision of the mechanisms behind oil-water (O/W) emulsion preparation by ultrasound and cavitation. Ultrason. Sonochem..

[23] Tang S.Y., Shridharan P., Sivakumar M. (2013). Impact of process parameters in the generation of novel aspirin nanoemulsions -Comparative studies between ultrasound cavitation and microfluidizer. Ultrason. Sonochem..

[24] Jadhav A.J., Holkar C.R., Karekar S.E., Pinjari D.V., Pandit A.B. (2015). Ultrasound assisted manufacturing of paraffin wax nanoemulsions: Process optimization. Ultrason. Sonochem..

[25] Raviadaran R., Ng M.H., Manickam S., Chandran D. (2019). Ultrasound-assisted water-in-palm oil nano-emulsion: Influence of polyglycerol polyricinoleate and NaCl on its stability. Ultrason. Sonochem..

[26] Zhu Y., Chen T., Meng Z., Li T., Zhang J., Zhang N., Luo G., Wang Z., Zhou Y. (2025). Preparation and characterization of a novel green cinnamon essential oil nanoemulsion for the enhancement of safety and shelf-life of strawberries. Int. J. Food Microbiol..

[27] Nie Y., Pan Y., Jiang Y., Xu D., Yuan R., Zhu Y., Zhang Z. (2023). Stability and bioactivity evaluation of black pepper essential oil nanoemulsion. Heliyon.

[28] Jeong M.-S., Cho S.-J. (2024). Effect of pH-shifting on the water holding capacity and gelation properties of mung bean protein isolate. Food Res. Int..

[29] Hel S.E., Mokhtar S.M. (2011). Chemical composition and nutritive value of lantana and sweet pepper seeds and nabak seed kernels. J. Food Sci..

[30] Yılmaz E., Hüriyet Z. (2017). Physico-Chemical and Functional Properties of Extracted Capia Pepperseed (*Capsicum annuum* L.) Proteins. Waste Biomass Valor..

[31] Liao L., Liu T.-X., Zhao M.-M., Cui C., Yuan B.-E., Tang S., Yang F. (2010). Functional, nutritional and conformational changes from deamidation of wheat gluten with succinic acid and citric acid. Food Chem..

[32] Yi H., Talmon G., Wang J. (2019). Glutamate in cancers: From metabolism to signaling. J. Biomed. Res..

[33] Razak M.A., Begum P.S., Viswanath B., Rajagopal S. (2017). Multifarious Beneficial Effect of Nonessential Amino Acid, Glycine: A Review. Oxid. Med. Cell. Longev..

[34] Ruszczyńska M., Sytkiewicz H. (2024). New Insights into Involvement of Low Molecular Weight Proteins in Complex Defense Mechanisms in Higher Plants. Int. J. Mol. Sci..

[35] Kong J., Yu S. (2007). Fourier transform infrared spectroscopic analysis of protein secondary structures. Acta Bioch. Bioph. Sin..

[36] Barth A. (2007). Infrared spectroscopy of proteins. BBA Bioenerg..

[37] Carbonaro M., Nucara A. (2010). Secondary Structure of Food Proteins by Fourier Transform Spectroscopy in the Mid-Infrared Region. Amino Acids.

[38] Moure A., Sineiro J., Domínguez H., Parajó J.C. (2006). Functionality of oilseed protein products: A review. Food Res. Int..

[39] Benelhadj S., Gharsallaoui A., Degraeve P., Attia H., Ghorbel D. (2016). Effect of pH on the functional properties of Arthrospira (Spirulina) platensis protein isolate. Food Chem..

[40] Tang Q., Roos Y.H., Miao S. (2023). Plant Protein versus Dairy Proteins: A pH-Dependency Investigation on Their Structure and Functional Properties. Foods.

[41] Shen Y., Tang X., Li Y. (2021). Drying methods affect physicochemical and functional properties of quinoa protein isolate. Food Chem..

[42] Zhang Q., Chen Y., Liu W., Ye Y., Cheng D., Zheng H., Wu L. (2025). Effects of Tweens on the Structure, interfacial Characteristics, and emulsifying and foaming properties of Ovalbumin. Food Res. Int..

[43] Pan Z., Trusler J.M., Jin Z., Zhang K. (2025). Interfacial property determination from dynamic pendant-drop characterizations. Nat. Protoc..

[44] Xu J., Zhu X., Zhang J., Li Z., Kang W., He H., Wu Z., Dong Z. (2023). Nanoemulsification of soybean oil using ultrasonic microreactor: Process optimization, scale-up and numbering-up in series. Ultrason. Sonochem..

[45] Belgheisi S., Motamedzadegan A., Milani J.M., Rashidi L., Rafe A. (2021). Impact of ultrasound processing parameters on physical characteristics of lycopene emulsion. J. Food Sci. Technol..

[46] Yang G., Lin W., Lai H., Tong J., Lei J., Yuan M., Zhang Y., Cui C. (2021). Understanding the relationship between particle size and ultrasonic treatment during the synthesis of metal nanoparticles. Ultrason. Sonochem..

[47] Chen J., Dickinson E. (1998). Viscoelastic properties of protein-stabilized emulsions: Effect of protein-surfactant interactions. J. Agric. Food Chem..

[48] Albano K.M., Fazani C.Â.L., Nicoletti V.R. (2019). Electrostatic interaction between proteins and polysaccharides: Physicochemical aspects and applications in emulsion stabilization. Food Rev. Int..

[49] Ren M.H., Du Q.H., Li J.M., Liu X.L., Wei L.S., Lan H.X., Fu Z. (2025). Emulsion-stabilizing properties of Moringa oleifera seed protein and chitosan: Impact of molecular weight and protein concentration. J. Food Meas. Charact..

[50] Yu M.J., Feng R., Long S., Tao H., Zhang B. (2025). Stabilizing emulsions by ultrasound-treated pea protein isolate-tannic acid complexes: Impact of ultrasonic power and concentration of complexes on emulsion characteristics. Food Chem..

[51] Pratap-Singh A., Guo Y., Lara Ochoa S., Fathordoobady F., Singh A. (2021). Optimal ultrasonication process time remains constant for a specific nanoemulsion size reduction system. Sci. Rep..

[52] Kowarit S., Sathapornprasath K., Jansri S.N. (2024). Application of hot air-derived RSM conditions and shading for solar drying of avocado pulp and its properties. Sol. Energy.

[53] Yang Q., Qi W., Shao Y., Zhang X., Wu F., Zhang Z. (2024). Stability and pH-Dependent mechanism of Astaxanthin-Loaded nanoemulsions stabilized by almond protein isolate. Foods.

[54] McClements D.J., Gumus C.E. (2016). Natural emulsifiers-Biosurfactants, phospholipids, biopolymers, and colloidal particles: Molecular and physicochemical basis of functional performance. Adv. Colloid Interface Sci..

[55] Tan Y., Lee P.W., Martens T.D., McClements D.J. (2022). Comparison of emulsifying properties of plant and animal proteins in oil-in-water emulsions: Whey, soy, and RuBisCo proteins. Food Biophys..

[56] Liu W.Y., Feng M.Q., Wang M., Wang P., Sun J., Xu X.L., Zhou G.H. (2018). Influence of flaxseed gum and NaCl concentrations on the stability of oil-in-water emulsions. Food Hydrocoll..

[57] Tian Y., Taha A., Zhang P., Zhang Z., Hu H., Pan S. (2021). Effects of protein concentration, pH, and NaCl concentration on the physicochemical, interfacial, and emulsifying properties of β-conglycinin. Food Hydrocoll..

[58] Ma W., Wang J., Wu D., Chen H., Wu C., Du M. (2020). The mechanism of improved thermal stability of protein-enriched O/W emulsions by soy protein particles. Food Funct..

